# Case of needle tract seeding during preoperative neoadjuvant chemotherapy for resectable pancreatic cancer

**DOI:** 10.1002/deo2.124

**Published:** 2022-05-09

**Authors:** Kei Yane, Mai Aoki, Yusuke Tomita, Masahiro Yoshida, Kotaro Morita, Hideyuki Ihara, Tetsuya Sumiyoshi, Hitoshi Kondo, Yumiko Oyamada

**Affiliations:** ^1^ Department of Gastroenterology Tonan Hospital Hokkaido Japan; ^2^ Department of Pathology Tonan Hospital Hokkaido Japan

**Keywords:** endoscopic ultrasonography, EUS‐FNA, neoadjuvant chemotherapy, pancreatectomy, pancreatic cancer

## Abstract

Reports of needle tract seeding (NTS) as a complication of endoscopic ultrasound‐guided fine‐needle aspiration (EUS‐FNA) have been increasing. To date, most of the reported cases of NTS have been diagnosed during the postoperative follow‐up period. Herein, we report a case of NTS that occurred during preoperative neoadjuvant chemotherapy after EUS‐FNA for resectable pancreatic cancer. The patient underwent transgastric EUS‐FNA for a pancreatic tail tumor. He was diagnosed as having resectable pancreatic cancer and received preoperative neoadjuvant chemotherapy. After completion of the chemotherapy, computed tomography showed a thick‐walled cyst‐like structure appearing between the pancreas and the gastric wall. Combined resection revealed adenocarcinoma invasion into the cyst‐like structure. Based on the clinical course, and surgical and pathological findings, the condition was diagnosed as NTS. It is thus crucial that after EUS‐FNA, a detailed review of the imaging findings be conducted in the preoperative period. If adhesions between the stomach and the pancreas are observed after transgastric EUS‐FNA, combined resection of the gastric wall should be considered.

## INTRODUCTION

Preoperative neoadjuvant chemotherapy has been recently reported to prolong recurrence‐free survival and overall survival in resectable pancreatic cancer. A recent randomized trial of neoadjuvant chemotherapy in Japan (Prep‐02/JSAP05) also suggests the use of preoperative neoadjuvant chemotherapy with gemcitabine and S‐1 for resectable pancreatic cancer.^1^ Thus, the importance of preoperative histopathological diagnosis is increasing. Endoscopic ultrasound‐guided fine‐needle aspiration (EUS‐FNA) is widely used for the histopathological diagnosis of solid pancreatic lesions with high sensitivity and specificity.^2^ In their recent large multicenter retrospective study, Kanno et al. reported that the EUS‐FNA‐related adverse events in histopathologic diagnoses were not severe conditions and had a low incidence.^3^ However, preoperative transgastric EUS‐FNA for pancreatic cancer occasionally results in needle tract seeding (NTS) observed after surgery. Although NTS after EUS‐FNA has been regarded as an extremely rare adverse event, it has been increasingly reported and cannot be ignored.^4^, ^5^, ^6^, ^7^ Most reported NTS cases have been diagnosed during the postoperative follow‐up period. However, NTS during preoperative neoadjuvant chemotherapy for borderline resectable pancreatic cancer has been reported recently.^8^ Herein, we report a case of NTS occurring during preoperative neoadjuvant chemotherapy after EUS‐FNA for resectable pancreatic cancer.

## CASE REPORT

**FIGURE 1:**
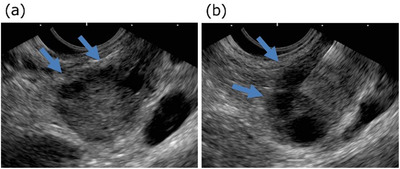
(a) Endoscopic ultrasound showed a 20‐mm‐diameter low‐echoic tumor in the pancreatic tail. There were multiple cystic lesions at the tumor margins, which were thought to be dilated branched pancreatic ducts (arrows). (b) A transgastric puncture was performed with a 22‐G Franseen needle (Acquire, Boston Scientific). The puncture site was close to the dilated branched pancreatic ducts (arrows)

The patient was an 82‐year‐old man with worsening diabetes mellitus. Abdominal ultrasonography showed a pancreatic mass, and he was referred to our hospital for detailed examination. He had been taking aspirin because of a history of angina pectoris. Computed tomography (CT) showed a 22‐mm‐diameter low‐density mass in the pancreatic tail. The dynamic study showed a delayed enhancement pattern. The distal main pancreatic duct was dilated. A blood test showed an elevated CA19‐9 level of 652 U/ml. Pancreatic cancer was suspected, and EUS‐FNA was performed to confirm the diagnosis. EUS showed a 20‐mm‐diameter low‐echoic tumor in the pancreatic tail. There were multiple cystic lesions at the tumor margins, which were thought to be dilated branched pancreatic ducts. A transgastric puncture was performed with a 22‐G Franseen needle (Acquire, Boston Scientific), with 20 ml aspiration. Although there was no aspiration of pancreatic juice and no fluid collection around the tumor on EUS during the procedure, the puncture site was close to the dilated branched pancreatic ducts (Figure 1). The cytology was positive/malignant, and the histological diagnosis was suspicious for adenocarcinoma. There were no postprocedural adverse events. The lesion was diagnosed as resectable pancreatic cancer, cT3N0M0, and cStageIIA (UICC 8th).

**FIGURE 2:**
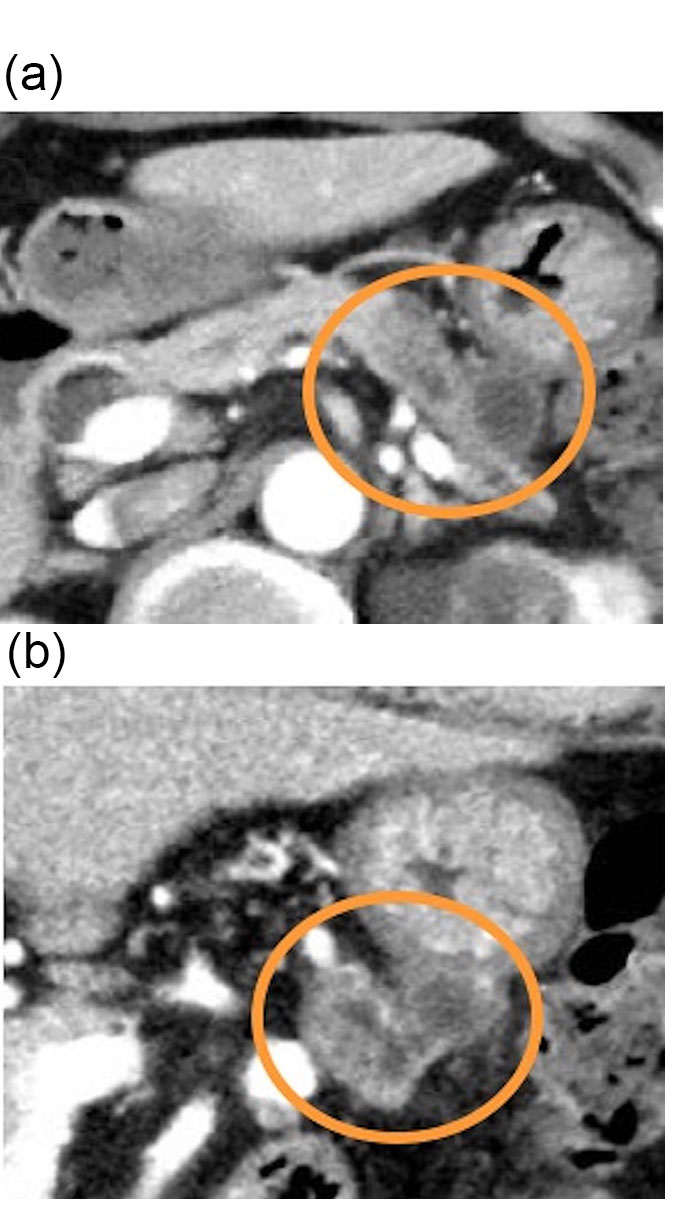
(a) Axial image and (b) coronal image computed tomography revealed a new 18‐mm‐diameter cystic lesion with a thick wall between the pancreas and the stomach (circle)

**FIGURE 3:**
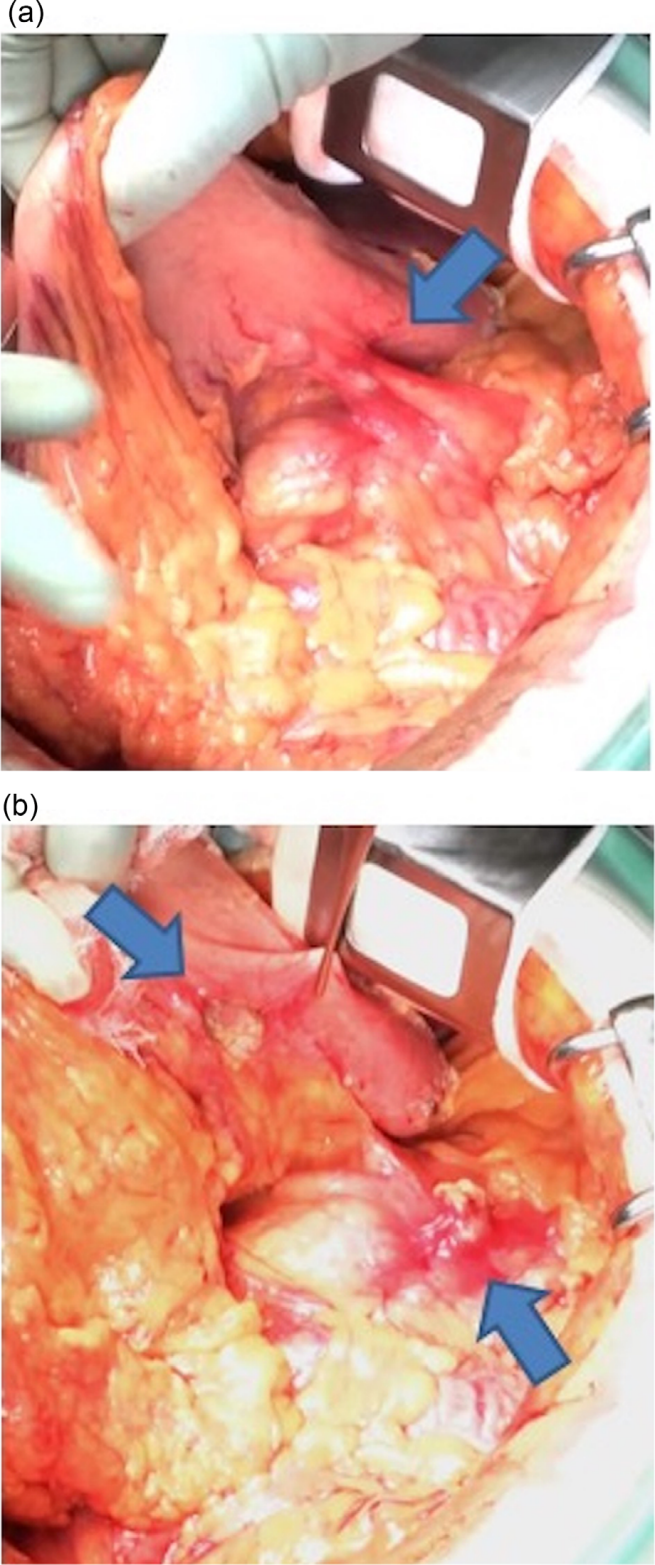
(a) A strong adhesion was observed between the posterior wall of the stomach and the anterior surface of the pancreas (arrow) and (b) partial resection of the gastric wall was performed (arrows)

After surgical consultation, preoperative neoadjuvant chemotherapy (gemcitabine and S‐1 therapy) was started. The patient received intravenous gemcitabine at 1000 mg/m^2^ on days 1 and 8, and S‐1 orally at 80 mg/m^2^ on days 1–14 of a 21‐day cycle. The treatment was repeated for two cycles. The duration of the preoperative neoadjuvant chemotherapy was 6 weeks. After two courses of chemotherapy, a CT scan was performed for detailed evaluation. Although there was no increase in the tumor size, a thick‐walled cyst‐like structure appeared between the pancreas and the gastric wall, which was not observed on the CT before chemotherapy (Figure 2). There were no obvious unresectable factors such as invasion of the celiac trunk / superior mesenteric arteries or distant metastasis. The CA19‐9 level was 1420 U/ml before starting the chemotherapy and was 1053 U/ml at the end of the chemotherapy. Based on the clinical course and location, a localized encapsulated pancreatic fistula formed after EUS‐FNA was suspected. As there were no signs of infection, the patient underwent a distal pancreatectomy as planned. The duration from EUS‐FNA to surgery was 90 days. There was no obvious peritoneal dissemination. Surgical findings showed firm adhesions between the posterior wall of the stomach and the anterior surface of the pancreas, and a partial combined resection of the gastric wall was performed (Figure 3).

**FIGURE 4:**
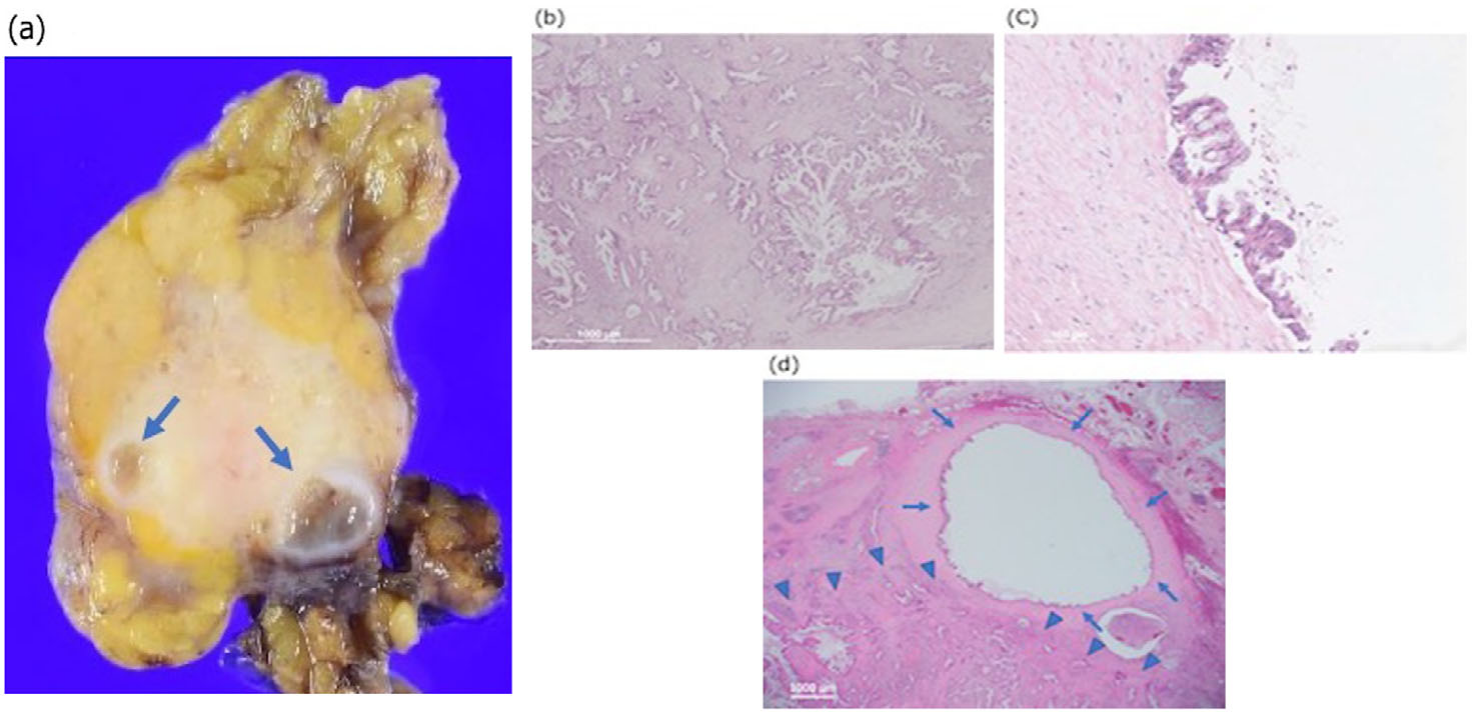
(a) Multiple cystic structures were observed at the tumor margins (arrows), (b) the main tumor was a well‐differentiated adenocarcinoma (×20), (c) cystic structures were lined by epithelium with mild to moderate atypia (×200), and (d) there was no continuity between the cystic structures (arrows) and the main tumor (arrowheads; ×20)

**FIGURE 5:**
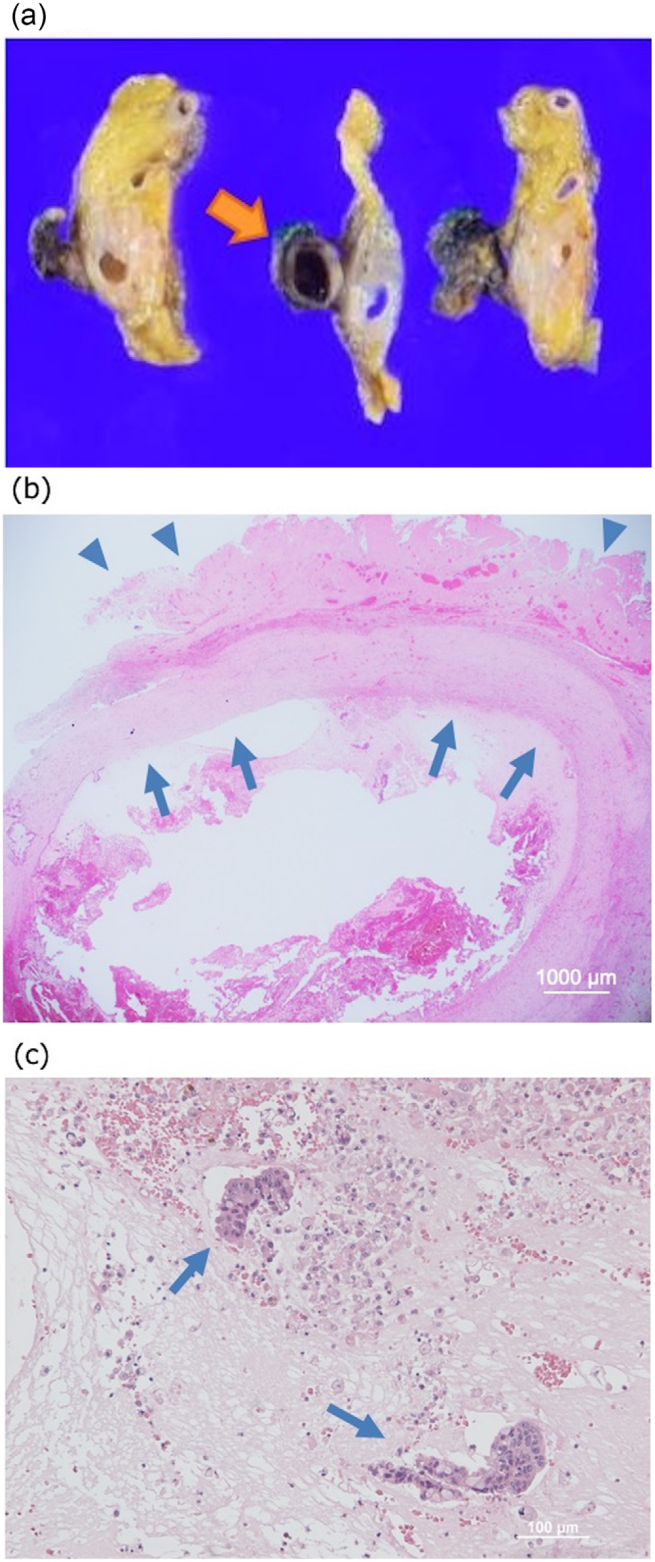
(a) Cystic lesion with a thick wall on the surface of the pancreas was observed (arrow), (b) histopathological examination revealed a round‐shaped connective tissue (arrows) between the resected gastric wall (arrowheads) and the anterior surface of the pancreas (×12.5), and (c) adenocarcinoma invasion was observed in the connective tissue (arrows; ×100)

Gross examination of the resected specimen revealed a white, irregularly shaped tumor in the pancreatic tail. On the caudal side of the tumor, there was dilatation of the main pancreatic duct and atrophy of the pancreatic acinus, stromal fibrosis, and inflammatory cell infiltration, which were findings of obstructive pancreatitis associated with pancreatic duct obstruction. Pathologically, the main tumor was a well‐differentiated adenocarcinoma measuring 30 × 22 × 15 mm^3^. There were multiple cystic structures at the tumor margins, which were lined by epithelium with mild to moderate atypia. There was no continuity between the cystic structures and the main tumor (Figure 4). There was no lymph node metastasis. Adenocarcinoma invasion was observed in the round‐shaped connective tissue between the resected gastric wall and the anterior surface of the pancreas (Figure 5). There was no continuity with the main tumor. Based on the clinical course, and surgical and pathological findings, the condition was diagnosed as NTS after EUS‐FNA. The pancreatic resection margins and the surrounding dissected surface were both negative for cancer. The histological response to the chemotherapy was Grade I with Evans classification. The final stage of the disease was pT3N0M0, pStage IIA. The postoperative clinical course was uneventful, and the patient was discharged 22 days later. S‐1 adjuvant chemotherapy was performed and the patient is currently under follow‐up. It is now 12 months after the operation, and the patient has not had any cancer recurrence.

## DISCUSSION

To our knowledge, this is the first report of NTS during preoperative neoadjuvant chemotherapy for resectable pancreatic cancer.

Recently, the number of reports on NTS after EUS‐FNA for pancreatic cancer has been increasing. To date, all NTS cases have undergone transgastric EUS‐FNA for pancreatic body or tail cancer. Additionally, most NTS cases have been diagnosed during the postoperative follow‐up period. The mean interval from EUS‐FNA to NTS detection was previously reported as 22 months (range 1–67 months).^5^ Recently, with the development of preoperative chemotherapy, a case of NTS during long‐term (8 months) chemotherapy for locally advanced pancreatic cancer extending to the celiac plexus has been reported.^8^

Although the mechanisms and risk factors of NTS have not yet been clarified, most reports to date have involved multiple punctures, and an increase in the number of punctures could theoretically increase the risk of NTS.^5^ However, in the present case, NTS was observed even though only one puncture was performed.

Another factor that should be considered for NTS occurrence in the present case based on the CT image changes before and after EUS‐FNA is the possibility that subclinical pancreatitis or pancreatic juice leakage occurred after the puncture. In this case, the puncture site was close to the dilated branched pancreatic ducts, and damage to the branched pancreatic ducts may have caused the leakage of pancreatic juice. Additionally, the puncture route passed near the dilated main pancreatic duct, and although no aspiration of pancreatic juice was observed during the procedure, we cannot completely rule out the possibility that the needle may have injured the main pancreatic duct. A case of a pancreatic fistula occurring after FNA and resulting in NTS has been reported.^9^ Such a case may have a similar mechanism to our case.^9^ We believe that it is important to avoid puncturing the main pancreatic duct and dilated branched pancreatic ducts to prevent pancreatic fluid leakage from EUS‐FNA.

Regarding NTS treatment, complete resection may reportedly contribute to longer survival.^5^, ^6^ Therefore, if adhesions between the stomach and the pancreas are observed during surgery as in our case, it is suggested to perform a combined resection of the gastric wall considering the possibility of NTS.^10^ If suspected during surgery, an R0 resection may be achieved by performing a combined resection of the gastric wall.

With the JSAP‐02 report, preoperative neoadjuvant chemotherapy is now recommended and widely used even in resectable pancreatic cancer. Thus, tissue diagnosis by EUS‐FNA is becoming increasingly important. Consequently, the number of cases similar to the present case may increase in the near future.

It is important to conduct a detailed review of the imaging findings for any changes suspicious of NTS during the preoperative period. Additionally, if adhesions between the stomach and the pancreas are observed during surgery after transgastric EUS‐FNA, combined resection of the gastric wall should be considered.

## CONFLICT OF INTEREST

The authors declare that they have no conflict of interest.

## ETHICS STATEMENT

Not applicable.

## FUNDING INFORMATION

Not applicable.

## References

[1] Motoi F , Kosuge T , Ueno H *et al*. Randomized phase II/III trial of neoadjuvant chemotherapy with gemcitabine and S‐1 versus upfront surgery for resectable pancreatic cancer (Prep‐02/JSAP05). Jpn J Clin Oncol 2019; 49: 190–4. 30608598 10.1093/jjco/hyy190

[2] Banafea O , Mghanga FP , Zhao J , Zhao R , Zhu L . Endoscopic ultrasonography with fine‐needle aspiration for histological diagnosis of solid pancreatic masses: A meta‐analysis of diagnostic accuracy studies. BMC Gastroenterol 2016; 16: 108. 27580856 10.1186/s12876-016-0519-z PMC5007683

[3] Kanno A , Yasuda I , Irisawa A *et al*. Adverse events of endoscopic ultrasound‐guided fine‐needle aspiration for histologic diagnosis in Japanese tertiary centers: Multicenter retrospective study. Dig Endosc 2021; 33: 1146–57. 33284491 10.1111/den.13912

[4] Yane K , Kuwatani M , Yoshida M *et al*. Non‐negligible rate of needle tract seeding after endoscopic ultrasound‐guided fine‐needle aspiration for patients undergoing distal pancreatectomy for pancreatic cancer. Dig Endosc 2020; 32: 801–11. 31876309 10.1111/den.13615

[5] Gao RY , Wu BH , Shen XY *et al*. Overlooked risk for needle tract seeding following endoscopic ultrasound‐guided minimally invasive tissue acquisition. World J Gastroenterol 2020; 26: 6182–94. 33177792 10.3748/wjg.v26.i40.6182 PMC7596640

[6] Kitano M , Minaga K , Hatamaru K , Ashida R . Clinical dilemma of endoscopic ultrasound‐guided fine needle aspiration for resectable pancreatic body and tail cancer. Dig Endosc 2022; 34: 307–16. 34437750 10.1111/den.14120

[7] Park JS , Lee JH , Song TJ *et al*. The impact of preoperative EUS‐FNA for distal resectable pancreatic cancer: Is it really effective enough to take risks? Surg Endosc 2022; 36: 3192–9. 34254183 10.1007/s00464-021-08627-3

[8] Matsumoto K , Kato H , Tanaka N , Okada H . Preoperative detection of tumor seeding after endoscopic ultrasonography‐guided fine needle aspiration for pancreatic cancer. Intern Med 2018; 57: 1797–8. 29434140 10.2169/internalmedicine.0321-17 PMC6047977

[9] Okamoto T , Nakamura K , Takasu A , Kaido T , Fukuda K . Needle tract seeding and abscess associated with pancreatic fistula after endoscopic ultrasound‐guided fine‐needle aspiration. Clin J Gastroenterol 2020; 13: 1322–30. 32720220 10.1007/s12328-020-01188-3

[10] Matsui T , Nishikawa K , Yukimoto H *et al*. Needle tract seeding following endoscopic ultrasound‐guided fine‐needle aspiration for pancreatic cancer: A report of two cases. World J Surg Oncol 2019; 17: 134. 31382964 10.1186/s12957-019-1681-x PMC6683495

